# Physicochemical, Microbiological and Sensory Evaluation of Plant-Based Meat Analogs Supplemented with Phenolic Extracts from Olive Mill By-Products

**DOI:** 10.3390/foods14193347

**Published:** 2025-09-26

**Authors:** Adina Nichita, Beatrice Sordini, Ebtsam Al-Olayan, Sonia Esposto, Egidia Costanzi, Beniamino Cenci-Goga, Mona Elena Popa, Maurizio Servili, Gianluca Veneziani

**Affiliations:** 1 Faculty of Biotechnologies, University of Agronomic Sciences and Veterinary Medicine of Bucharest, 59 Măraști Blvd, District 1, 011464 Bucharest, Romania; nichitaadina1979@gmail.com (A.N.); monapopa@agral.usamv.ro (M.E.P.); 2 Department of Agricultural, Food and Environmental Sciences, University of Perugia, via S. Costanzo, 06126 Perugia, Italy; sonia.esposto@unipg.it (S.E.); maurizio.servili@unipg.it (M.S.); gianluca.veneziani@unipg.it (G.V.); 3 Department of Zoology, College of Science, King Saud University, Riyadh 11451, Saudi Arabia; eolayan@ksu.edu.sa; 4 Department of Veterinary Medicine, University of Perugia, 06126 Perugia, Italy; egidia.costanzi@unipg.it (E.C.); beniamino.cencigoga@unipg.it (B.C.-G.); 5 Department of Paraclinical Sciences, Faculty of Veterinary Science, University of Pretoria, Onderstepoort 0110, South Africa

**Keywords:** meat analogs, olive mill vegetation water, microbiological analysis, phenolic compounds, bioactive properties, antioxidant activity, sensory analysis, shelf life, sustainability, food preservation, circular economy

## Abstract

The bioactive properties of a phenolic extract (PE) obtained from olive mill vegetation water (OVW) in powder formulation were utilized to enrich a meat analog composed of lentils and champignon mushrooms. The primary phenolic compounds in this extract were oleacein, verbascoside, and hydroxytyrosol. The effects on the final product were assessed over eight days of storage at 4 °C ± 2 under 12 h of light. The control samples were compared with two meat analogs enriched with ascorbic acid (AA) at 5 g kg^−1^ and one enriched with PE at 30 g kg^−1^. The physicochemical parameters (pH, aw, color, texture, and total phenol content), antioxidant activity, microbial assessment, and sensory evaluations of meat analog samples were evaluated at three different time points (T0, T4, T8) during shelf life. The PE-enriched meat analogs maintained a relatively high and stable phenolic concentration throughout their shelf life, significantly enhancing the antioxidant activities of the final product. The addition of PE also influenced the growth of *Enterococcus* spp., *Lactococcus* spp., and *Lactobacillus* spp. during storage. The results of the triangular test indicated perceptible differences between AA and PE meat analogs. Meanwhile, the quantitative descriptive analysis (QDA) emphasized notable enhancements in odor and texture characteristics for PE-enriched samples. Plant-based meat analogs can benefit from the effective use of PE (antioxidant and sensory properties), supporting the sustainable reuse of olive oil by-products.

## 1. Introduction

The agriculture and food industries play essential roles in both the European and global economies [1]; however, they also pose significant challenges related to the management of waste and food losses [2]. The absence of effective waste management practices in raw material processing and product manufacturing results in large volumes of waste or by-products that negatively impact the environment [3]. The olive oil sector produces a large amount of solid (olive pomace and leaves) and liquid (OVW) waste, the quantity of which depends greatly on the different two- or three-phase technology extraction process. Only in the Mediterranean area is the OVW annual production estimated to be between 10 and 30 million m^3^ [4,5]. Adopting zero-waste or circular economy approaches could help mitigate these negative impacts by minimizing waste generation and promoting sustainable practices throughout the food industry.

Currently, functional foods are a growing market segment that requires useful bioactive ingredients [6,7,8,9,10]. Recently, special attention has been given to natural compounds derived from plant extracts and their association with valuable bioactivity [11]. The negative impacts of animal production drive technological advancements in the development of alternatives, such as protein-based meat analogs. These alternatives provide a more sustainable and eco-friendly option for consumers who wish to reduce their environmental footprint while still enjoying familiar flavors and textures in their diets [12,13,14]. Plant-based meat analogs are textured food products made from plant proteins that aim to replace or mimic meat [15]. Textured plant proteins, the primary ingredients in these analogs, are typically extruded from a mixture of soy protein, wheat gluten, pea protein, and other ingredients [16]. Meat analogs often contain more than 20 ingredients, including fats, sugars, vitamins, minerals, phosphates, organic acids, and others [17]. While extrusion technology is commonly used to produce these products, other methods can also be employed [18].

Plant proteins possess a well-balanced amino acid profile and have excellent potential to replace meat by facilitating the development of healthy meat-like products that are high in protein, low in saturated fat, cholesterol-free, and nutritionally comparable to traditional meats and meat preparations [19]. However, many plant-based proteins are deficient in at least one essential amino acid, such as lysine, methionine, or cysteine [19]. Mushrooms, for example, offer a high protein content comparable to that of animals or poultry, and they are on par with soybean and pea protein and higher than those of wheat [20]. Additionally, olive mill vegetation water (OVW) contains phenolic compounds, including phenolic acids, phenolic alcohols, flavonoids and secoiridoids [9]. An effective way to sweeten the acceptance of plant-based meat alternatives is to consume hybrid products that combine meat and plant-based ingredients at a 50:50 ratio, especially during a transition phase [21]. The development of functional foods with added fiber and antioxidants has shown positive health effects. Increased intake of fiber and antioxidants of natural origin is associated with protection against cardiovascular disease, metabolic syndrome and chronic illnesses [22]. Replacing synthetic additives with natural alternatives increases dietary fiber and antioxidant intake and improves the shelf life and rheological properties of supplemented products [23].

A recent study highlighted the use of apple pomace as a valuable food ingredient for enhancing the nutritional and antioxidant properties of Italian salami [6]. Furthermore, fruits, vegetables and other foodstuffs are rich sources of dietary carbohydrates and bioactive phytochemical compounds that provide essential nutrition and significant health benefits [24]. Roila et al. [25] investigated the effects of a phenolic extract from OVW as a natural additive with antioxidant and antimicrobial properties on fresh beef burgers compared with and without synthetic additives. A further study reported that incorporating green tea leaf powder into lamb salami effectively inhibited bacterial growth and lipid oxidation, although it did not improve sensory quality [26].

To the best of our knowledge, this study is the first to explore the use of phenolic extract (PE) derived from olive mill vegetation water (OVW) as an ingredient to increase the microbiological, physicochemical, and sensory properties of plant-based meat analogs during refrigerated storage. The efficacy of this natural extract as a substitute for synthetic preservatives in plant-based meat analogs was investigated. Specifically, the physicochemical parameters, antioxidant activity, antimicrobial properties, and sensory characteristics of the OVW phenolic extract in powder formulation were evaluated and compared to those of a control formulation (without additive) and a control formulation containing a synthetic additive (ascorbic acid) over an 8-day period of refrigerated storage. PE presents a promising opportunity as a “clean label” ingredient, aligning with the increasing consumer demand for food products that prioritize safety and health benefits. Moreover, sourcing PE from OVW offers a sustainable approach to minimizing food waste while enhancing the value of a by-product that is typically highly polluting.

## 2. Materials and Methods

### 2.1. Chemicals, Reagents and Materials

Pure analytical standards for the phenolic compounds: tyrosol (*p*-HPEA; ≥98.0%), hydroxytyrosol (3,4-DHPEA; ≥90.0%), and verbascoside (≥99%) were purchased from Merck (Milan, Italy), whereas oleacein (3,4-DHPEA-EDA) and oleocanthal (*p*-HPEA-EDA) were obtained as described by Taticchi et al. [27]. The chemical compounds 2,4,6-tripyridyl-s-triazine (TPTZ), 2,2-diphenyl-1-picrilidrazil (DPPH˙), phosphotungstic phosphomolybdic acid (Folin–Ciocalteu’s reagent), anhydrous sodium carbonate and all the other chemical compounds and solvents were supplied by Merck (Milan, Italy).

The ingredients for preparing the plant-based meat analog were purchased from a local supermarket (Perugia, Italy). PE was obtained from OVW and dried by Extracta S.n.c. (Muggio (MB), Italy), according to the protocol reported by Mercatante et al. [28].

Microbiological analysis: Maximum recovery diluent (MRD), Oxoid, Basingstoke, Hampshire, UK), plate count agar (PCA, Oxoid), Man, Rogosa and Sharpe agar (MRS, Oxoid), M17 agar (Oxoid), Enterococcus agar (ENT, Oxoid), Baird-Parker agar (BP, Oxoid) containing egg yolk tellurite (Oxoid), violet red bile glucose agar (VRBG, Oxoid), violet red bile lactose agar (VRBL, Oxoid), and Pseudomonas agar base (PS103, Oxoid) solutions were prepared in the laboratory of the Department of Agricultural, Food and Environmental Sciences, University of Perugia.

### 2.2. Production of Plant-Based Meat Analogs

**Figure 1:**
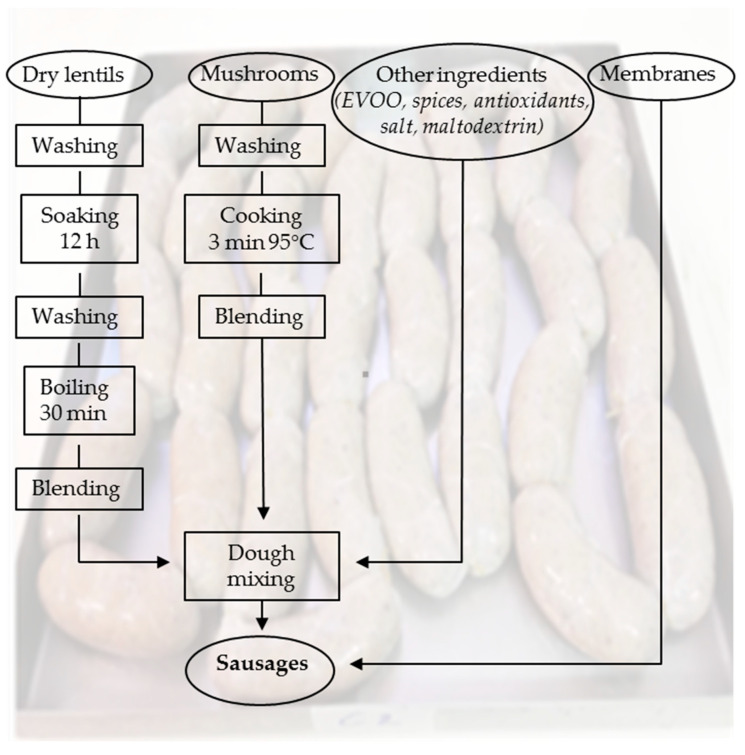
Flow chart of plant-based meat analogs production.

The samples of the meat analogs were produced from lentils (300 g kg^−1^), champignon mushrooms (350 g kg^−1^), extra virgin olive oil (10 g kg^−1^), spices (pepper, garlic and paprika), and salt and antioxidants (ascorbic acid and phenolic extract), as detailed in Table 1 and Figure 1. The dry lentils were washed with cold water and left to soak for 12 h. The outer membrane of the lentils was cleaned by repeated washing in cold water and boiling (30 min). After boiling, the lentils were drained via a salad spinner to remove excess water, cooled and processed via a mincer. The mushrooms were also thoroughly washed, drained of excess water, and cooked for 3 min at 95 °C. After cooking, the mushrooms were cooled and ground using a mincer. A total of ten kilograms of meat analog dough were collected and divided into three equal parts for three different formulations: a control sample (additive-free), a sample with the addition of ascorbic acid (5 g kg^−1^), and a sample with the addition of PE (30 g kg^−1^) obtained from OVW. An equal amount of 15 g of maltodextrin present in 30 g of PE was included in the C and AA samples. Additionally, extra virgin olive oil and spices were added, and the doughs were accurately mixed to ensure good homogeneity of the ingredients.

Finally, each of the three distinct doughs was placed into a membrane, yielding a total of approximately 30 sausages (approximately 100 g each) of plant-based meat analogs for each formulation, as illustrated in Figure S1. The plant-based meat analog samples were stored in a cold room maintained at 4 ± 2 °C for 8 days under 12 h of light exposure (600 lx). The room temperature was monitored via a portable device TESTO 175-T2 (Testo, Lenzkirch, Germany). Ten sausages were sampled for each formulation at the beginning of storage (T0) and after 4 and 8 days of storage (T4 and T8).

### 2.3. Phenolic Extract (PE) from Olive Vegetation Water (OVW)

Within 24 h of the virgin olive oil extraction process carried out with a three-phase decanter, OVW was treated with 0.5 L/ton of enzymatic preparation at room temperature for 12 h. As described by Servili et al. [29], PE was obtained via a green technology based on tangential membrane filtration (microfiltration, ultrafiltration and reverse osmosis) that can produce a phenolic concentrate (PC). Spray-dry technology was used to stabilize the PE, which produced a phenolic powder rich in bioactive molecules, mainly oleuropein derivatives (oleacein and hydroxytyrosol) and verbascoside. The phenolic extract powder was produced by Extracta S.n.c. (Muggio (MB), Italy).

#### Phenolic Composition

HPLC analysis of PE phenolic compounds was conducted as outlined by Mercatante et al. [28]. Briefly, 0.2 g of PE was dissolved in 10 mL of water. The solution was then filtered via a 0.2 µm polyvinylidene fluoride (PVDF) syringe filter (Agilent Captiva, Agilent Technologies, Santa Clara, CA, USA) before being injected into an HPLC instrument (Agilent Technologies system Model 1100 (Agilent Technologies, Santa Clara, CA, USA)) equipped with a diode array detector (DAD) and fluorescence detector (FLD). The HPLC equipment and analytical conditions were consistent with those previously described by Selvaggini et al. [30]. Each measurement was conducted in duplicate, and the data are expressed in mg g^−1^ of PE.

### 2.4. Physicochemical Analysis of the Meat Analog

#### 2.4.1. Bromatological Parameters

##### Moisture Content

The moisture content of the raw meat analogs was determined according to method B described in ISO 662 [31]; the method was adapted to the specific working conditions. Approximately 2 g of the meat analog samples were distributed on the surfaces of the aluminum capsules and placed in an oven (Binder GmbH, Tuttlingen, Germany) at 105 ± 2 °C for 24 h. The samples removed from the oven were placed in a desiccator until constant values were obtained and subsequently weighed. Two replicates for each sample were carried out.

##### Ash Content

The ash was evaluated via the official method of the official and tentative methods of the American Oil Chemists’ Society [32]. This method results in ash residue remaining after incineration under the conditions specified for the test. A platinum capsule with a capacity of 25 mL was passed through the flame to remove any residues and placed in a desiccator for 3 h until the temperature stabilized, after which the sample was weighed. Approximately 2 g of dry sample was placed in a platinum capsule and then placed in a muffle oven preheated to 600 ± 15 °C for two hours. The capsule was transferred to a desiccator until the temperature stabilized, and the sample was weighed to determine the ash content. Two replicates for each sample were carried out.

##### Lipid Content

The lipid content of the meat analogs was analyzed via a Soxhlet extractor, where 10 g of the meat analog sample (s.u.) with 5 g of punch was loaded into a thimble made of thick filter paper and placed inside the main compartment of the Soxhlet extractor. The process took approximately six hours, using n-hexane as the extraction solvent, and the solvent was removed via a rotary evaporator, Rotavapor R-210 (Buchi Italia s.r.l Cornaredo, Italy). The residual oil content was detected according to the official methods of analysis [33]. Two replicates for each sample were carried out.

#### 2.4.2. pH and Water Activity

The physicochemical and texture analyses of the meat analogs were performed according to the protocol described by Grispoldi et al. [6]. The pH of the samples was measured with an S40 Seven-Multi digital pH meter (Mettler-Toledo Italia, Novate Milanese, Italy) after mixing 10 g of meat analog with 90 mL of distilled water and then homogenizing with a stomacher for one minute. A HygroLab 3 dew point hygrometer (Rotronic, Huntington, NY, USA) was used to measure water activity (Aw). Two replicates for each sample were carried out.

#### 2.4.3. Texture Analysis

The hardness of each sample was measured via an FL 100 digital dynamometer (Sauter Italia, Cinisello Balsamo, Milan, Italy). A modified version of the Warner–Bratzler shear force method was used: all samples were cut into cylinders 2 cm high and 1 cm in diameter; three cylinders were used to measure the resistance to the application of a compressive force (applied by using a flat dynamometer head), whereas the other three were used to measure the resistance of the sample to the application of a shear force (applied with a head in wedge shape), both with a head speed of 250 mm/min. Two replicates for each sample were carried out.

#### 2.4.4. Color Analysis

The “ColorMeter RGB Colorimeter” app (White Marten GmbH, Stuttgart, Germany) was used to measure color of the samples using an iPhone XS running iOS 13.7, as described by the authors in previously published works [23]. The conventional colorimeters (such as the one described below) are designed to determine the color of a single point in a uniform area. In this case, we chose to measure the average color of the sample in order to replicate the consumer’s perception. The “ColorMeter RGB Colorimeter” app was calibrated against a reference colorimeter, a Minolta CR 200 Chroma Meter (Konica Minolta Inc. Tokyo, Japan). Briefly, the Minolta CR 200 Chroma Meter was used to measure a series of red/reddish calibration plates (specifically, the CR-A47 DP, CR-A47 R, and CR-A47 B) in conjunction with a standard white plate in order to determine the CIELAB L* (lightness), a* (redness), and b* (yellowness) color spaces, and the results were used to calibrate the readout of the “ColorMeter RGB Colorimeter” app. The Minolta CR 200 Chroma Meter was set to measure under the CIE Standard Illuminant D65. D65 is approximately equivalent to the average midday light in Western Europe/Northern Europe, which includes both direct sunlight and diffused light from a clear sky. Hence, it is also referred to as a daylight illumination and has a correlated color temperature of approximately 6500 K. The light used to illuminate the calibration plates for the “ColorMeter RGB Colorimeter” app was, therefore, a source of 6500 K light (Godox Led 64, Godox, Shenzhen, China) under controlled conditions in a photographic light box. The CIELAB system describes colors visible to the human eye according to their hue and chroma (position on the a* and b* axes) and their lightness, L*, which corresponds to a position on a black-to-white scale.

#### 2.4.5. HPLC Analysis of Phenolic Compounds from OVW

Phenols were extracted from plant-based meat analogs, as reported by Miraglia et al. [34], with some modifications. Five grams of each sample were dissolved in 50 mL of a methanol/water mixture (80:20 (*v*/*v*), homogenized with a blender (Osterizer Blender Cycle Blend Pulse 4153-50, Sunbeam Products, Inc., Boca Raton, FL, USA) for 1 min, and then centrifuged at 9000 rpm for 10 min. The methanolic supernatant was recovered and collected in a 250 mL rotavapor flask. The extraction was repeated two times. The solvent was evaporated under vacuum at 36 °C via a rotary evaporator, and the obtained residue was brought to 25 mL with distilled water to obtain an aqueous extract rich in phenolic compounds (AEs). The AE was analyzed via the same HPLC instrument (Agilent Technologies, Santa Clara, CA, USA) and the conditions described above in Section Moisture Content. The quantitative and qualitative concentrations of phenolic compounds derived from olive vegetation water and extracted from meat analogs (PEs) during shelf life were expressed in g kg^−1^ dry weight. Two replicates for each sample were carried out.

#### 2.4.6. Antioxidant Activity

The antioxidant activity of the meat analogs was evaluated via a 2,2-diphenyl-1-picrilidrazil (DPPH˙) assay according to the method proposed by Brand-Williams et al. [35]. The DPPH˙ solution was prepared by accurately weighing 12.5 mg of DPPH˙ and bringing it to volume in a 50 mL volumetric flask with methanol, followed by a 10-fold dilution. DPPH˙ reagent (3.8 mL) was added and mixed with 200 µL of AE from each plant-based meat analog sample with a dilution ranged from 0 to 50 times. Measurements were obtained using a Cary 100 Scan UV-Visible Spectrophotometer (Varian, Walnut Creek, CA, USA), and the results were expressed as μmol TROLOX equivalent (TE) per g of dry weight based on a TROLOX calibration curve [36]. Three replicates for each sample were carried out.

#### 2.4.7. Total Phenols

The total phenol content (TPC) was determined according to the authors’ description via colorimetric methods [37]. Briefly, 0.4 mL of AE was dissolved in 1.6 mL of water, then reacted with 8 mL of Folin–Ciocalteu (FC) reagent diluted tenfold, followed by neutralization with 10 mL of anhydrous sodium carbonate solution (7.5% *w*/*v*). After a 2 h incubation, the absorbance was measured at 765 nm using a Cary 100 Scan UV-Visible spectrophotometer (Varian, Walnut Creek, CA, USA). Calibration was performed using gallic acid as a standard, and results were expressed as g of gallic acid per kg of sample according to previously reported by Esposto et al. [38]. Three replicates for each sample were carried out.

### 2.5. Microbiological Analysis

The methodology used for microbiological analysis has already been described by the authors in a previous work [39]. Briefly, for each analysis, 25 g of meat was homogenized in a sterile bag with 225 mL of peptone water (PW, Oxoid, Basingstoke, Hampshire, UK) by using a stomacher. Tenfold dilutions were prepared by using sterile tubes with 9 mL of maximum recovery diluent (MRD, Oxoid). The dilutions were inoculated in triplicate on different culture media. For each analysis, eight bacterial populations were evaluated: total aerobic mesophilic flora, *Lactobacillus* spp., *Lactococcus* spp., enterococci, *Staphylococcus* spp., *Enterobacteriaceae*, total coliforms and *Pseudomonas* spp. The methodology used for the microbiological analysis has already been described by the authors in a previous work [40]. The number of colonies was then converted to a log of colony-forming units per gram (log cfu g^−1^), and the mean was calculated for each analysis. The sensitivity for the spread plate was 10^2^ CFU/g, that for the pour plate was 10 CFU/g, and the 95% confidence limit was set between ±37% and ±12% (i.e., plates with a number of CFUs ranging from 30 to 300). Therefore, all plates with less than 30 CFUs were not used for data analysis, and when this was applied to the lowest dilution, the results were recorded as <300 for the pour plate and <3000 for the spread plate [41].

### 2.6. Sensory Evaluations

To assess whether differences among plant-based meat analog samples (C, AA, and PE) could be detected on sampling days T0, T4, and T8, triangle tests were performed following the ISO guidelines [42]. A panel of 18 experts, consisting of staff and students from the University of Perugia, Italy, balanced in gender and appropriately trained according to ISO [43], were recruited based on their experience in sensory evaluation of various foods. The triangle tests and result calculations adhered to the methodology outlined by Dottori et al. [44]. During the three triangle test sessions, each panelist participated in three rounds representing the difference test comparisons (C vs. AA, C vs. PE, and AA vs. PE), with the sampling order randomized for each participant. Three samples were presented per round and coded differently, with two identical and one differing. For each round, panelists were required to identify the different samples; even if they were uncertain, a response was mandatory (forced choice). Sensory sheets were collected for each round to record the number of correct and incorrect responses. Statistical analysis was conducted via the table on Page 433 of Meilgaard et al. [45], with the null hypothesis of “no difference” between samples differently formulated being rejected if the number of correct responses was greater than or equal to the tabled value for 18 observations (α ≤ 0.01). The minimum number of correct responses required for significance was 12. Three sensory evaluation sessions were conducted at different sampling times in individual booths in a control environment (22 °C) under regular white tube lighting. The samples, approximately 2 cm in diameter, were cut into 2 cm pieces to resemble commercial product sizes. Each tasting session was accompanied by an instruction sheet indicating the sample codes and the specified tasting sequence. Owing to the unavailability of real-time microbiological analysis results and to exclude potential health risks, the panelists were instructed to evaluate the samples based solely on visual appearance, smell, and texture without tasting them. Each evaluation session was held between 10 a.m. and 12 p.m.

In addition to the triangle test evaluations, quantitative descriptive sensory analysis (QDA) was conducted on the plant-based meat analog samples (C, AA, and PE) in accordance with the ISO standard [41]. The QDA involved 10 expert panelists (4 men and 6 women, aged between 25 and 55) trained to recognize and quantify the sensory characteristics of plant-based meat analogs. As with the triangle test, three sensory evaluation sessions were conducted at each sampling time (T0, T4, and T8), during which the panelists assessed the meat analogs based on various attributes, including appearance (red color, brown color, green/gray color, color homogeneity, firmness (V), presence of casing, oiliness, and brightness), odor (overall odor, specific odors (starchy, leguminous, mushroom, fresh vegetable, garlic, spices, oil, and metallic)), texture (hardness, springiness, juiciness, softness, clumpiness, gumminess, cohesiveness, firmness (T), fat content (oiliness-greasiness), and moisture) overall pleasantness, and off-odor (rancid and anomalous fermentation). Ratings were assigned using an unstructured 9 cm scale for each descriptive attribute. Sensory evaluations were performed on individual booths according to ISO standard [41]. As mentioned, the samples were cut into cylinders measuring 2 cm × 2 cm, labeled with a three-digit code and served to the panelists in random order.

All participants involved in the sensory evaluations (triangular test and QDA) at various sampling times were informed about the study’s compliance with ethical standards (WMA Declaration of Helsinki) and provided informed consent prior to participating in the visual and olfactory assessments of plant-based meat analogs, made of ingredients listed in Table 1.

### 2.7. Statistical Analysis

Bromatological and physicochemical analyses were performed in duplicate and microbiological analyses in triplicate; results are expressed as mean ± standard deviation (SD). Statistical evaluation of total phenolic content (TPC), phenolic composition, and antioxidant activity was carried out with SigmaPlot v.12.5 (Systat Software Inc., San Jose, CA, USA). The evolution of phenolic composition was studied using one-way ANOVA with Tukey’s test (*p* < 0.05) to identify significant differences among samples over the course of 8 days of storage. Additionally, a two-way analysis of variance (ANOVA) was used to analyze the antioxidant activity (DPPH˙) and total phenol content (TPC) data, setting formulation (F), storage time (ST) as factors, and their interaction (F × ST). A post hoc Tukey’s honest significance test at a 95% confidence level (*p* < 0.05) was used.

Microbiological data were analyzed with GraphPad Prism v.6.0h for Mac OS X (GraphPad, San Diego, CA, USA) using the same statistical approach, considering product type as the treatment factor. A *p*-value < 0.05 was considered statistically significant. Sensory data from quantitative descriptive analysis (QDA) were elaborated by principal component analysis (PCA) with Panel Check software v.1.2.1 (Nofima, Norway).

## 3. Results and Discussion

The phenolic compounds of olive fruit were recovered by using a filtration system for the purification and concentration treatment of OVWs obtained by the extraction of virgin olive oil with microfiltration, ultrafiltration and reverse osmosis membranes [29]. Spray-dry technology was used to stabilize the PE, which produces a phenolic powder rich in bioactive molecules [9], mainly oleuropein derivatives (oleacein and hydroxytyrosol), verbascoside and lower amounts of ligstroside derivatives (oleocanthal and tyrosol), as shown in Table 2. Maltodextrin, a common drying agent, was used for the production of PE powder. This inert carrier of the spray drying technology was also appropriately included at the same dose in the other two plant-based doughs to reduce the possibility of a variable that could have determined eventual interferences during microbiological growth and shelf life of the product.

### 3.1. Bromatological and Physicochemical Characteristics

The plant-based meat analogs, which are made mainly of lentils and mushrooms (Table 1), are characterized by moisture, lipid and protein contents of approximately 69%, 3% d.w. and 27% d.w., respectively, which can be considered appreciable values from a nutritional point of view and characterized by an energy of about 117 Kcal/100 g following the nutritional database of the USDA [46]. The Bromatological and physicochemical parameters at T0 of the storage period (Table 3) were very similar among the three different trials. The only exception was the slight decrease in the pH value of the AA sample due to the addition of 5 g kg^−1^ ascorbic acid and the increase in the ash content of the PE sample, which was probably due to the enrichment with 30 g kg^−1^ phenolic powder from OVW.

Moreover, the moisture content of the two plant-based meat analogs enriched with antioxidant powder also varied with decreasing humidity during the preparation of sausage dough, with values of 7.1% and 11% for the AA and PE samples, respectively. The different meat analogs resulted in the following changes in moisture content during the storage period: C, 69.0%, 69.0% and 68.4%; AA, 64.1%, 63.0% and 59.8%; and PE, 61.4%, 61.3 and 54.4%, at 0, 4 and 8 days of storage, respectively (Table 4). The moisture content did not vary in the control test over 8 days of refrigerated storage. In contrast, both AA and PE were stable until 4 days of storage and then decreased to 6.7% and 11.4%, respectively, at the end of storage (Table 4).

The pH values were stable for the AA samples during the refrigeration period; otherwise, the control test and the meat analog enriched with PE slightly decreased at T8 of storage (Table 5).

From a physical point of view, the analysis of color through the evaluation of the CIELAB coordinates (Table 5) revealed slight variability among the three different trials. The slight variability found among the C, AA, and PE samples across storage times suggests that neither ascorbic acid nor phenolic extract dramatically alters overall color stability compared to the control. The luminosity and yellow–blue coordinates of the C and AA meat analogs seemed to decrease at T4 and then increase again at the end of the shelf life. The observed decrease and subsequent recovery of luminosity (L*) and yellow–blue (b*) coordinates in C and AA samples may be attributed to minor moisture loss or limited pigment oxidation during storage. These changes seem to be temporary and correct themselves by the end of shelf life, indicating a degree of resilience in the color profile of these formulations. As supposed by Varga-Visi et al. [47], analyzing the color evolution of sausages with paprika stored under refrigerated conditions, the increase in L* could be due to the loss of pigments of the raw material contributing to brighter sausages. This effect might be faster in plant-based meat analogs due to the presence of a higher quantity of different classes of pigments when compared with a meat product. The degradation of natural pigments could be slowed down by the addition of ascorbic acid during the shelf life confirming the lower L* value of AA samples when compared with the control test at the end of storage time. In contrast, the PE samples tended to be more uniform, with decreases and increases in L* and b*, respectively, during the storage period, which was probably due to the oxidation of phenolic compounds of OVW, which increased the natural browning process of the sausage raw material. So, the browning effect could cover and contrast the increase in lightness detected in the other two samples of plant-based meat analog (C and AA). In addition, as reported by other authors [48], the higher reduction in moisture content might also play an important role in the decrease in lightness of the final product. While these changes can mimic natural sausage browning, they might also affect visual appeal depending on consumer preferences. This behavior is consistent with the literature, where phenolics in plant matrices are known to contribute to non-enzymatic browning during storage [49]. The red–green coordinates were very low in all the tests and tended to slightly decrease during shelf life. The measured numbers of all trials during the storage time were on the positive half-axis, which measures redness (a*). Even if the observed decline in redness was very low and not statistically significant, this effect could be due to the deterioration of paprika carotenoids such as capsanthin and capsorubin during the shelf life [47,48,50]. The consistently low and slightly decreasing a* values across all trials highlight that these plant-based sausages lack strong red pigment retention, which could be a limitation if replicating the look of traditional meat products is desired. The minimal decrease over shelf life, however, implies these formulations are relatively stable in this attribute.

The data on the compression and shear force (Table 5) indicated a similar texture for the three different meat analogs, indicating good structural stability, with a slight increase in hardness for the PE sample at the end of refrigerated storage, in accordance with the lowest moisture content at T8 of conservation. This suggests that the PE may promote or coincide with moisture loss or greater matrix cross-linking, resulting in a firmer product. This could represent a beneficial or negative aspect depending on marked requirements and consumers’ sensory perception that influences attitudes and preference.

Others by-products belonging to different food technologies were applied to the meat-analogs production sector mainly to improve the texture with a right integration of fiber [51,52,53] or to gain other characteristics to enhance health, nutritional and sensory properties of the final product such as antioxidants, natural colorants, vegetable oils, minerals, and vitamins, with a particular attention to shelf life and food safety [54,55].

Collectively, the minor color and texture differences over the storage life indicate that phenolic extract can be incorporated without drastically altering key physical attributes. However, their subtle effects—particularly the browning and textural changes shown with the phenolic extract—should be carefully considered depending on desired product characteristics and target consumer preferences. In fact, as reported by Kyriakopoulou et al. [56], several studies are concerning with the possibility of addition of different ingredients and additives to produce meat-like texture and color in an attempt to achieve its sensorial and nutritional aspects and meet the needs of different categories of consumers.

### 3.2. Phenolic Content and Antioxidant Activity

The AEs obtained from the different samples of plant-based meat analogs were analyzed with FC reagent to evaluate the total phenols derived from the raw materials, vegetables and spices and from the enrichment of the products with PE from OVW.

The analysis of total phenols did not reveal any significant changes in concentration for each different meat analog during the 8 days of shelf life at refrigerated temperature, except for samples AA and PE at T8 (Table 6). The slight variability of the data during storage could still be due to the particle size and homogeneity of the raw material and of the entire sausage dough, which are common characteristics of this type of foodstuff. Considering that the colorimetric method used is influenced by the reaction of protein with the FC reagent [57], the analysis revealed the highest values for the AA samples, but the data were obviously not due to a relatively high concentration of phenolic compounds but rather to the presence of ascorbic acid, which also reacts with the reagent [58]. The addition of PE improved the phenolic concentration of the meat analog, with a value approximately 3.2, 3.1, and 2.3 times greater than that of the control samples at each sampling time. The total phenol content showed good stability until the 4th day of storage, with a significant decrease (−20.3%) after 8 days of storage.

Table 7 shows the results of the HPLC analysis for the evaluation of the main phenolic compounds of OVW during the shelf life of the meat analogs enriched with PE. At T0, the phenolic content extracted from the PE samples was 468.6 mg kg^−1^. This value is much lower than the phenolic molecules included in the product, highlighting the possible reduction in the bioactive molecules for the oxidation process that can occur during the preparation of the sausage dough. However, the main cause of the reduction in recovered phenols from meat analogs at T0, equal to 42.8%, was probably due to the chemical interactions between bioactive phenolic molecules and the food matrix. As reported by other authors [59,60], many molecules are able to interact with polyphenols, such as proteins, which results in permanent chemical bonds with phenolic compounds, reducing their extractability and antioxidant power. De Toffoli et al. [61] reported high amounts of nonextractable phenolic compounds in olive mill wastewater for bean purée, among other plant-based foods. Even in this study, the leguminous proteins of lentils probably interacted with polyphenols, as the most reactive compound, determining protein aggregation, was oleacein. In contrast, verbascoside showed the lowest binding affinity and was, in fact, the molecule with the highest extractability from the raw material. During shelf life, the phenolic concentration decreased by 35.1% and 40.2% at T4 and T8, respectively (Table 7). The greatest reduction involved the oleacein content, which was not detected at T4 or T8, or the hydroxytyrosol content. In contrast, verbascoside and tyrosol seem to be more stable during the refrigeration period and less susceptible to oxidation when included in plant-based foods.

The data on free radical scavenging evaluated through the DPPH˙ assay confirmed the results of the analysis carried out with the FC reagent and the HPLC analysis of secoiridoid derivatives and verbascoside, which revealed the same trend during the shelf life of the product (Table 6). The AA meat analog presented the highest values, which were stable at the end of the storage period. In contrast, the PE samples significantly decreased under refrigerated conditions in the cold room, with decreases of 15% and 31% at T4 and T8, respectively. The antioxidant activity of the PE samples was greater than that of the control samples at all the storage times evaluated. PE meat analogs were characterized by values 5.6, 6.3, and 4.2 times greater than those of the corresponding C samples at T0, T4 and T8, respectively.

The data on both phenolic compounds and antioxidant activity at the end of the storage period highlighted the importance of enriching the meat analogs with bioactive molecules extracted from OVW that are able to guarantee a higher quality of the foodstuff. In fact, the bioactive molecules of PE meat analogs were also present after a cooking process on a stovetop grill pan, with a reduction in the content of approximately 10–25% of the total HPLC phenols [62]. The presence of these phenolic molecules is involved in improving the health characteristics of the final product due to the potential activities of the major OVW biophenols extracted from PE samples, such as antioxidant, cardioprotective, antiatherogenic, anti-inflammatory, antimicrobial, and chemopreventive activities [9,63]. In fact, oleuropein derivatives such as oleacein and hydroxytyrosol are the same molecules that lend health claims to the polyphenols of virgin olive oil, a pillar of the Mediterranean diet [64].

### 3.3. Microbiological Results

For the evaluation of bacterial populations during the shelf life of the products, statistically significant differences (*p* < 0.05) were observed for the total aerobic mesophilic microbiota on Day 2 (C vs. AA and PE) and for *Lactobacillus* spp. on Day 4 (AA vs. PE). For *Enterococcus* spp. and *Lactococcus* spp., no significant differences were observed (Table 8). The bacterial load of these groups at the beginning of the process was approximately 5 log CFU g^−1^, whereas at the end of storage at 4 °C, it reached values above 10^8^ cfu g^−1^. On average, these bacterial populations reached relatively high values in the control batches (C). This can be attributed to the mild antibacterial activity of ascorbic acid (AA) and phenolic compounds (PEs). For the spoilage bacterial populations, statistically significant differences were observed for *Pseudomonas* spp. on Day 4, when the control groups (C) presented higher levels than did the AA and PE groups. The abundances of Enterobacteriaceae and coliform organisms on Day 8 were significantly lower in the AA groups. No significant differences were observed for *Staphylococcus* spp. In general, the bacterial concentration of Enterobacteriaceae, total coliforms and *Pseudomonas* spp. at the beginning of the process was approximately 5 log cfu g^−1^ and decreased during the shelf life for the AA groups but increased in the control and PE groups. *Staphylococcus aureus* was never detected during the experiment. These results show that ascorbic acid had some effect on Enterobacteriaceae, whereas phenolic compounds had no activity against spoilage microorganisms but adversely affected the growth of *Enterococcus* spp., *Lactococcus* spp., and *Lactobacillus* spp. The data reported against spoilage microorganisms or lactic acid bacteria is confirmed by some authors that analyzed different extracts from olive mill by-products showing an absence or low activity against different bacterial strains [65,66,67]. Even if, in some tests, the level of antimicrobial activity seems to be influenced by the concentration of the different phenolic compounds present in the polyphenolic extract. On the contrary, other studies, also applied to other food technological processes, showed an opposite antimicrobial effect [68,69,70,71]. The main secoiridoids derivatives (oleacein, oleochantal, hydoxytyrosol and tyrosol), phenolic acids and verbascoside, naturally present in the raw materials, such as table olives, or added as bioactive extracts to other products, highlighted a different effect on the growth of pathogenic and non-pathogenic microorganisms, probably influenced by microbial concentrations, physicochemical characteristics and nutrient availability of different food matrices [9,34,72,73,74]. In a future perspective of producing a fermented plant-based meat analog, as underlined by Fasolato et al. [66], the selective activity of lactic acid bacteria should be considered. In fact, it is a positive outcome, as these naturally occurring microbiota often negatively impact the quality of ripened salami by contributing to spoilage or undesirable fermentation profiles [75]. Lactic acid bacteria such as *Enterococcus* spp., *Lactococcus* spp., and *Lactobacillus* spp. are critical to the fermentation process of fermented products, but their overgrowth can sometimes lead to undesirable flavors or spoilage. Phenolic compounds can suppress the growth of *Lactobacillus* spp., *Enterococcus* spp., and *Lactococcus* spp., which are otherwise beneficial but can sometimes cause spoilage or off-flavors in the final product [76].

### 3.4. Sensory Evaluation

The use of various ingredients, flavors, and additives in plant-based meat analogs significantly impacts their sensory properties, especially their appearance and texture [77,78], and the relationships between instrumental texture variables and human perceptions of texture have been studied, with a focus on attributes such as hardness, cohesiveness, and fracturability. These sensory aspects are crucial factors that influence consumers’ decisions to choose these substitute foods over animal-derived foods [77,79,80].

In this study, the sensory evaluation of plant-based meat analogs (C, AA, and PE samples) during refrigerated storage was conducted via two distinct approaches: discriminatory testing (e.g., triangular tests) and descriptive sensory analysis (QDA). Table S1 presents the results of three triangular tests conducted by 18 expert panelists during the storage of plant-based meat analogs. The comparison between formulations AA and PE revealed significant differences at various sampling times (T0, T4, and T8). Furthermore, a significant difference was detected between C and PE at T0 and T4; however, no significant difference was detected between C and AA at T0.

**Figure 2:**
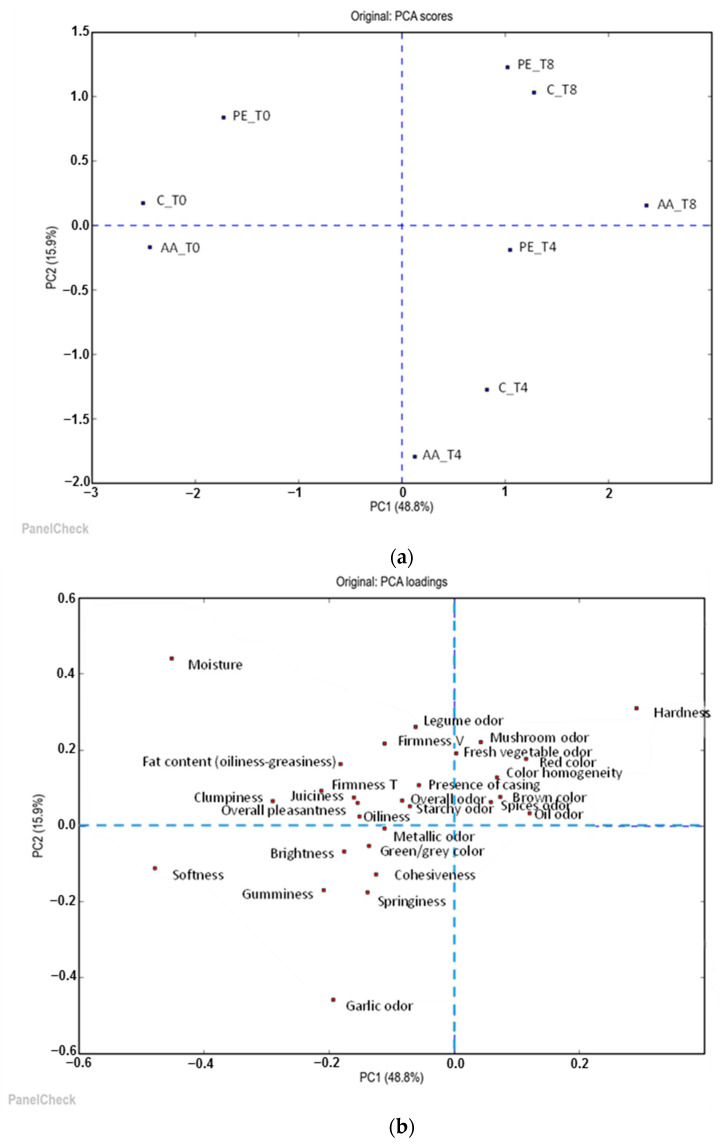
(**a**) Score plot and (**b**) loading plot of the first two principal components (PC1 vs. PC2) of the PCA model of sensory scores for the different plant-based meat analogs during 8 days shelf life.

To further investigate the differences among the three meat analog formulations, a trained panel of 10 panelists conducted a quantitative descriptive sensory analysis (QDA). The appearance, odor, and texture attributes as well as the flavors of the C, AA, and PE samples were evaluated at different sampling times (0, 4, and 8 days of storage). The collected data were analyzed via principal component analysis (PCA). The PCA model explained 90.8% of the total variance with five principal components, which accounted for the variance as follows: 48.8%, 15.9%, 12.6%, 8.5%, and 5.0%, respectively. Although five principal components were retained, explaining a cumulative 90.8% of the variance, PCs 3–5 did not contribute clear or relevant patterns and were therefore not further interpreted. For clarity, only PC1 and PC2 are discussed in detail. The score plot (PC1/PC2) clearly distinguishes the samples (C, AA, and PE) based on storage time, with samples at the beginning of storage (T0) positioned to the left of PC1, those after 4 days (T4) in the middle, and those at the end (T8) to the right of PC1 (Figure 2a). Within each storage time cluster, a clear differentiation based on formulation was observed along PC2, with PE samples positioned at the top, followed by C in the middle and AA at the bottom of the plot. The relative loading plot (PC1/PC2) (Figure 2b) shows that the variables most responsible for the distribution of the C, AA, and PE samples at the beginning of storage (on the left side of the figure) included moisture, softness, clumpiness, gumminess, firmness T, and garlic odor. These attributes are typically associated with plant-derived proteins, particularly soy protein, which contribute to meat analogs having a greater firmness T, gumminess, and chewy texture, as well as increased clumpiness due to their poor agglomeration properties [80]. Garlic is often included in plant-based meat analogs to mask the beany flavor, enhancing the overall flavor of these products [80]. In contrast to PC1, the samples of different formulations presented characteristics such as hardness, oil odor, red color, and color homogeneity at the end of refrigerated storage (Figure 2b). Among these attributes, hardness was the most significant factor influencing sample distribution. Several studies have reported an increase in hardness in plant-based meat analogs during storage, which is typically linked to changes in moisture content and interactions between ingredients, particularly protein, oil, and starch, over time [81]. The variables affecting the distribution of PE samples on the upper side of PC2 were moisture, hardness, legume odor, firmness V, and fresh vegetable odor. In contrast, the AA samples on the lower side of PC2 were characterized by garlic odor, gumminess, and springiness (Figure 2b). The control samples presented intermediate characteristics. The addition of PE might have masked the strong garlic odor that was prominent in the other samples. Several studies have examined the deodorizing effects of natural extracts, including mint leaves, rosemary, and parsley, on the odor of garlic in foods [82]. Conversely, the ascorbic acid intensified garlic’s odor, consistent with reports that it increases volatile sulfur levels by preventing their oxidation [83].

## 4. Conclusions

The valorization of bioactive molecules recovered from olive oil by-products and their use to fortify plant-meat analogs represents a possible way to carry out a circular economy and to sustain and improve the agri-food sector. The enrichment of phenolic compounds, such as oleacein, verbascoside and hydroxytyrosol, which are characterized by high antioxidant activities, guarantees high health properties to the final product during its shelf life. Compared with the control samples, the PE plant-based meat analogs presented greater values of total phenols and antioxidant power during 8 days of storage under refrigerated conditions and could represent a valid alternative to synthetic additives as ingredients of natural origin recovered via the use of green technologies.

The low extractable fraction of PE from the raw material, probably due to the interaction of polyphenols and other molecules, especially lentil proteins, could reduce its antioxidant and antimicrobial effects. Specifically, despite containing approximately 800 mg kg^−1^ of OVW-derived phenolic compounds, the PE showed no inhibitory activity against spoilage microorganisms. The possible strain selection that affects the growth of *Enterococcus* spp., *Lactococcus* spp., and *Lactobacillus* spp. should be considered and evaluated in a future perspective of being able to produce a fermented meat analog to avoid any alterations during the ripening and shelf life period.

From a sensory perspective, the PE samples were mainly characterized by higher moisture, hardness, firmness, and fresh vegetable and legume odors, together with a reduction in garlic odor, gumminess, and springiness compared to the other meat analogs. These sensory characteristics may contribute to a more balanced and appealing sensory profile, which could positively influence consumer acceptance of plant-based meat analogs enriched with PE.

## Figures and Tables

**Table 1:** Ingredients (g kg^−1^) of three different samples of plant-based meat analogs.

Samples	C	AA	PE
Dry lentils	300	300	300
Fresh champignon mushrooms	350	350	350
Extra virgin olive oil	10	10	10
Pepper	3	3	3
Fresh garlic	8	8	8
Paprika	2	2	2
Salt	12	12	12
Maltodextrin	15	15	-
Ascorbic acid	-	5	-
Phenolic extract	-	-	30

C = control, AA = ascorbic acid, PE = phenolic extract, the symbol “–“ indicates absence of ingredient.

**Table 2:** Phenolic composition (g kg^−1^) of PE recovered from olive mill vegetation water.

Phenolic Compounds	PE
Hydroxytyrosol (3,4-DHPEA) *	6.8 ± 0.03
Tyrosol (*p*-HPEA)	0.9 ± 0.02
Verbascoside	2.3 ± 0.06
Oleacein (3,4- DHPEA-EDA)	17.1 ± 0.6
Oleocanthal (*p*-HPEA-EDA)	0.3 ± 0.01
Total phenols	27.3 ± 0.6

* The data are the mean value of two samples analyzed two times ± standard deviation (*n* = 2).

**Table 3:** Bromatological and physicochemical parameters of plant-based meat analogs control (C), with ascorbic acid (AA) and with phenolic extract (PE).

Sample *	C	AA	PE
Moisture (%)	69 ± 0.7 a	64.1 ± 0.9 b	61.4 ± 0.5 b
Ash (% d.w.)	3.1 ± 0.1 a	3.1 ± 0.1 a	3.9 ± 0.3 b
Lipid (% d.w.)	3.2 ± 0.3 a	3.0 ± 0.3 a	3.0 ± 0.1 a
Total protein (% d.w.)	26.8 ± 1.4 a	26.7 ± 1.2 a	27 ± 1.6 a
pH	6.8 ± 0.0 a	5.8 ± 0.1 b	6.6 ± 0.1 a
Aw	96.2 ± 0.5 a	95.7 ± 0.1 a	95.4 ± 0.1 a

* The data are the mean values of two samples analyzed two times ± standard deviation (*n* = 2); d.w. = dry weight. The data in each row having different letters (a,b) are significantly different from one another (*p* < 0.05).

**Table 4:** Evolution of moisture content (%) during 8 days of shelf life at 4 °C ± 2 under 12 h of light condition (600 lx). Plant-based analogs: control (C); ascorbic acid (AA); phenolic extract (PE).

Time (Days) *	T0	T4	T8
C	69.0 ± 0.7 Aa	69.0 ± 0.9 Aa	68.4 ± 0.5 Aa
AA	64.1 ± 0.7 Ba	63.0 ± 0.6 Ba	59.8 ± 0.4 Bb
PE	61.4 ± 0.5 Ca	61.3 ± 0.3 Ca	54.4 ± 0.8 Cb

* The data are the mean values of two samples analyzed two times ± standard deviation (*n* = 2); For each different sample, the values having different capital letters are significantly different from one another (*p* < 0.05). For each different time, the values having different lower-case letters are significantly different from one another (*p* < 0.05).

**Table 5:** Evolution of physicochemical parameters during 8 days of shelf life at 4 °C ± 2 under 12 h of light condition (600 lx) (mean values ± SD, *n* = 2). Plant-based analogs: control (C); ascorbic acid (AA); phenolic extract (PE).

	C	AA	PE
**pH**			
day-0	6.79 ± 0.02 a	5.82 ± 0.02 b	6.6 ± 0.02 c
day-2	6.79 ± 0.02 a	6.12 ± 0.02 b	6.44 ± 0.02 c
day-4	6.81 ± 0.02 a	6.24 ± 0.02 b	6.53 ± 0.02 c
day-8	6.32 ± 0.02 ac	5.82 ± 0.02 b	6.32 ± 0.02 c
**a_w_**			
day-0	0.962 ± 0.005 ab	0.957 ± 0.001 a	0.954 ± 0.001 b
day-2	0.955 ± 0.002 ac	0.965 ± 0.001 b	0.96 ± 0.001 c
day-4	0.957 ± 0.008 a	0.955 ± 0.002 a	0.958 ± 0.001 a
day-8	0.957 ± 0.001 a	0.956 ± 0.004 ac	0.952 ± 0.001 c
**Shear force**			
day-0	3.18 ± 0.47 a	1.72 ± 0.29 b	2.00 ± 0.10 ab
day-2	1.12 ± 0.55 ab	1.57 ± 0.21 a	2.47 ± 0.20 b
day-4	4.58 ± 1.79 ab	4.72 ± 0.28 a	3.40 ± 0.38 b
day-8	5.28 ± 0.65 a	9.70 ± 2.75 a	6.35 ± 1.44 a
**Compression**			
day-0	11.70 ± 1.21 a	6.72 ± 1.00 b	6.60 ± 2.04 ab
day-2	4.85 ± 0.69 a	5.88 ± 0.26 a	8.02 ± 2.62 a
day-4	8.37 ± 0.65 a	17.55 ± 6.11 ab	15.30 ± 2.22 b
day-8	30.68 ± 4.85 a	25.78 ± 4.18 a	33.58 ± 8.18 a
L*			
day-0	52.67 ± 3.51 a	42.67 ± 2.89 b	44.67 ± 3.06 ab
day-2	34.33 ± 8.96 a	41.67 ± 1.16 a	39.33 ± 3.79 a
day-4	41.33 ± 1.16 a	40.67 ± 5.77 a	45.33 ± 1.53 a
day-8	56.00 ± 3.61 a	46.33 ± 1.53 a	40.33 ± 14.84 a
a*			
day-0	3.00 ± 1.00 a	5.33 ± 1.16 a	4.00 ± 1.00 a
day-2	3.33 ± 1.53 a	3.00 ± 1.00 a	3.33 ± 0.58 a
day-4	2.67 ± 1.16 a	4.67 ± 0.58 a	2.67 ± 1.53 a
day-8	2.00 ± 1.73 a	1.67 ± 1.16 a	1.33 ± 1.53 a
b*			
day-0	32.00 ± 1.73 a	35.00 ± 2.00 a	30.33 ± 5.03 a
day-2	26.33 ± 1.53 a	31.33 ± 0.58 b	31.67 ± 0.58 b
day-4	27.33 ± 3.22 a	29.33 ± 4.73 a	32.67 ± 4.51 a
day-8	38.67 ± 3.22 a	35.00 ± 0.00 a	33.00 ± 2.65 a

Different letters in the same row, for each time point, indicate means with statistically significant differences (*p* < 0.05). L* = lightness, a* = redness, b* = yellowness.

**Table 6:** Total phenol content (TPC) (g kg^−1^ d.w.) and antioxidant activity (DPPH˙) (μmol TE/g d.w.) of different plant-based meat analogs during 8 days shelf life.

		Storage Time (Days)						SEM	*p*		
	Samples	T0		T4		T8			F	ST	F × ST
Total phenol content (TPC)	C	0.85	Ca	0.84	Ca	0.94	Ca	0.12	<0.001	<0.05	<0.001
	AA	4.52	Ab	5.34	Aa	4.48	Ab				
	PE	2.75	Ba	2.58	Bab	2.19	Bb				
Antioxidant activity (DPPH˙)	C	1.82	Ca	1.39	Ca	1.69	Ca	0.67	<0.001	N.S.	<0.05
	AA	32.13	Aa	33.68	Aa	32.64	Aa				
	PE	10.21	Ba	8.72	Bab	7.05	Bb				

Results are reported as means and standard error means (SEM) of 3 replicates. Different capital letters indicate significant differences (Tukey’s test; *p* < 0.05) among formulations at the same storage time. Different lowercase letters indicate significant differences (Tukey’s test; *p* < 0.05) among storage times within the same formulation. C = control; AA = ascorbic acid; PE = phenolic extract; d.w. = dry weight; TE = Trolox equivalent; F = formulation; ST = storage time; N.S. = non-significant.

**Table 7:** Phenolic compounds (mg kg^−1^) of OVW detected in the meat analogs added with phenolic extract (PE) during shelf life.

Time (Days) *	T0	T4	T8
Hydroxytyrosol (3,4-DHPEA)	240.8 ± 3.1 a	205.2 ± 2.7 b	183.0 ± 5.7 c
Tyrosol (*p*-HPEA)	53.1 ± 1.1 a	52.5 ± 2.4 a	51.3 ± 1.9 a
Verbascoside	48.6 ± 2.9 a	46.7 ± 1.2 a	45.7 ± 1.7 a
Oleacein (3,4-DHPEA-EDA)	126.1 ± 8.0 a	0.0 ± 0.0 b	0.0 ± 0.0 b
*p*-HPEA-EDA	0.0 ± 0.0 a	0.0 ± 0.0 a	0.0 ± 0.0 a
Total phenols	468.6 ± 9.1 a	304.4 ± 3.9 b	280.0 ± 6.2 c

* The data are the mean values of two extractions evaluated two times ± standard deviation (*n* = 2). The data in each row having different letters (a–c) are significantly different from one another (*p* < 0.05).

**Table 8:** Results of microbiological analysis (log CFU g^−1^, mean values ± SD, *n* = 3).

	C	AA	PE
Mesophilic microbiota			
day-0	5.92 ± 0.27	5.01 ± 0.11	5.07 ± 0.36
day-2	6.38 ± 0.2 a	5.83 ± 0.16 b	5.51 ± 0.05 b
day-4	6.58 ± 0.37	5.37 ± 0.52	5.71 ± 0.46
day-8	8.85 ± 0.00 a	5.59 ± 0.83 b	8.24 ± 0.09 a
*Enterococcus* spp.			
day-0	<3	<3	<3
day-2	3.60 ± 0.00	<3	<3
day-4	4.90 ± 0.00	4.74 ± 0.00	<3
day-8	7.09 ± 0.55	6.78 ± 0.00	<3
*Lactococcus* spp.			
day-0	5.18 ± 0.33	4.95 ± 0.19	4.69 ± 0.02
day-2	5.64 ± 0.06	5.08 ± 0.52	5.71 ± 0.27
day-4	5.83 ± 0.68	5.85 ± 0.08	5.37 ± 0.09
day-8	8.08 ± 0.00	7.09 ± 0.13	7.78 ± 0.00
*Lactobacillus* spp.			
day-0	<3	<3	<3
day-2	4.45 ± 0.00	3.90 ± 0.00	3.00 ± 0.00
day-4	5.91 ± 0.00 ab	6.07 ± 0.06 a	4.57 ± 0.44 b
day-8	8.26 ± 0.00 a	8.11 ± 0.00 a	5.80 ± 0.06 b
*Staphylococcus* spp.			
day-0	<3	3.89 ± 0.58	3.70 ± 0.00
day-2	4.33 ± 0.21	5.05 ± 0.17	4.57 ± 0.13
day-4	3.00 ± 0.00	3.30 ± 0.00	<3
day-8	3.00 ± 0.00	3.48 ± 0.00	3.00 ± 0.00
*Pseudomonas* spp.			
day-0	4.06 ± 0.08	4.14 ± 0.20	3.89 ± 0.16
day-2	4.13 ± 0.18	4.09 ± 0.13	4.13 ± 0.18
day-4	6.82 ± 0.05 a	4.60 ± 0.14 b	5.69 ± 0.13 c
day-8	8.08 ± 0.00	7.90 ± 0.00	6.87 ± 0.04
Enterobacteriaceae			
day-0	5.30 ± 0.52	4.87 ± 0.15	4.91 ± 0.23
day-2	5.83 ± 0.50	5.30 ± 0.61	5.79 ± 0.08
day-4	6.78 ± 0.22	5.09 ± 0.55	6.78 ± 0.22
day-8	8.78 ± 0.00 ab	5.88 ± 0.36 a	8.30 ± 0.00 b
Coliform organisms			
day-0	5.20 ± 0.36	4.84 ± 0.17	4.76 ± 0.51
day-2	5.97 ± 0.45	5.45 ± 0.00	5.12 ± 0.16
day-4	6.21 ± 0.13 ab	5.07 ± 0.10 c	5.77 ± 0.21 b
day-8	8.24 ± 0.09 a	5.00 ± 0.00 b	7.15 ± 0.21 ab

Different letters in the same row, for each time point, indicate means with statistically significant differences (*p* < 0.05). No letters denote non-significant differences (*p* > 0.05).

## Data Availability

The original contributions presented in this study are included in the article/supplementary material. Further inquiries can be directed to the corresponding author.

## Supplementary Materials

The following supporting information can be downloaded at https://www.mdpi.com/article/10.3390/foods14193347/s1, Figure S1: Plant-based meat analogs. A: control (C); B: ascorbic acid (AA); C: phenolic extract (PE). Table S1: Triangle test sensory panel result (*n* = 18).
