# Supplementary material for: Physicochemical, Microbiological and Sensory Evaluation of Plant-Based Meat Analogs Supplemented with Phenolic Extracts from Olive Mill By-Products
Source: Foods. 2025 Sep 26;14(19):3347. doi: 10.3390/foods14193347 (PMC12523269; doi:10.3390/foods14193347)
Supplement: Supplementary file 1 [file foods-14-03347-s001.zip › Table S1.pdf]

Table S1. Triangle test sensory panel result (*n* = 18).

Days of Storage	Round	Number of Panelists	Number of Correct Answers	Number of Wrong Answers	$\alpha \leq 0.01$
T0	C vs. AA	18	15	3	Significant
T0	C vs. PE	18	9	9	Not significant
T0	AA vs. PE	18	16	2	Significant
T4	C vs. AA	18	3	15	Not significant
T4	C vs. PE	18	13	5	Significant
T4	AA vs. PE	18	15	3	Significant
T8	C vs. AA	18	11	7	Not significant
T8	C vs. PE	18	9	9	Not significant
T8	AA vs. PE	18	16	2	Significant
