# Supplementary figures and images for: Physicochemical, Microbiological and Sensory Evaluation of Plant-Based Meat Analogs Supplemented with Phenolic Extracts from Olive Mill By-Products
Source: Foods. 2025 Sep 26;14(19):3347. doi: 10.3390/foods14193347 (PMC12523269; doi:10.3390/foods14193347)

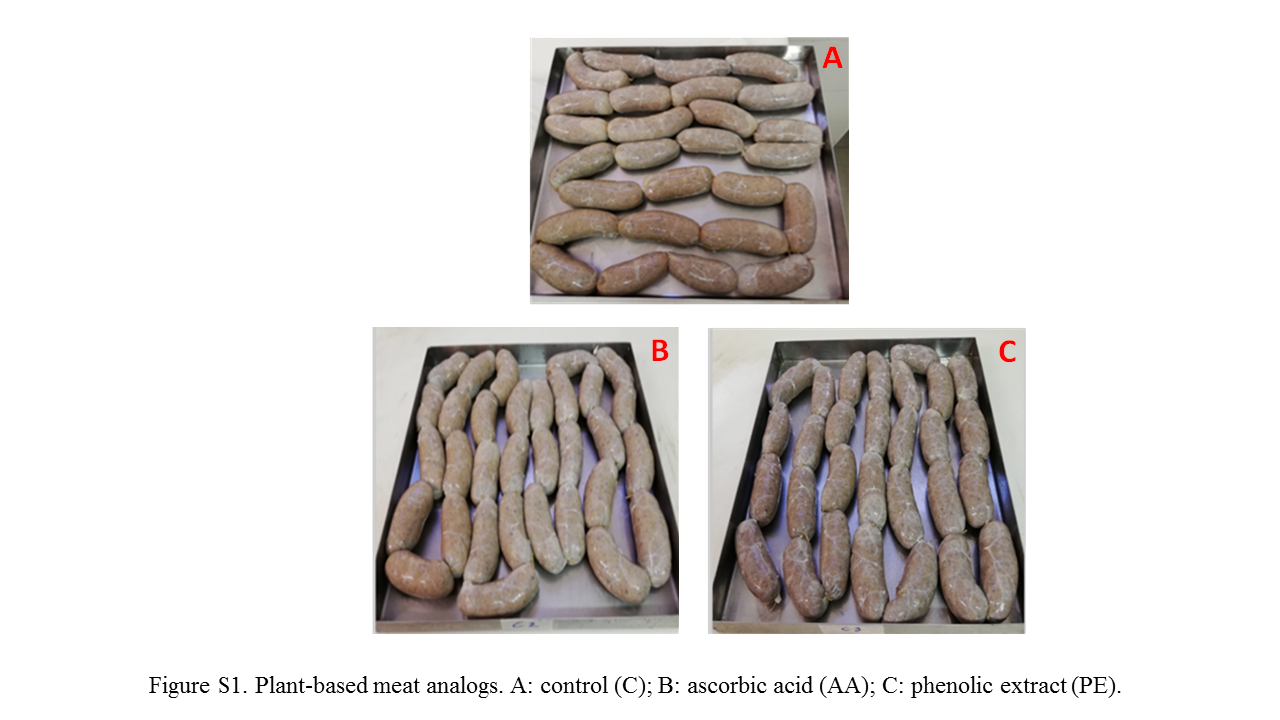

Supplement: Supplementary file 1 [file foods-14-03347-s001.zip › Figure S1.png]
